# Early childhood factors associated with obesity at age 8 in Vietnamese children: The Young Lives Cohort Study

**DOI:** 10.1186/s12889-021-10292-z

**Published:** 2021-02-05

**Authors:** Tuyen Nguyen, Karen Sokal-Gutierrez, Maureen Lahiff, Lia Fernald, Susan L. Ivey

**Affiliations:** 1 School of Public Health and School of Medicine, UC Berkeley-UCSF Joint Medical Program, University of California, 2121 Berkeley Way, Room 5302, Berkeley, CA USA; 2 School of Public Health, UC Berkeley-UCSF Joint Medical Program, University of California, 570 University Hall, MC 1190, Berkeley, CA USA; 3 Division of Epidemiology and Biostatistics, School of Public Health, University of California, 6132 Berkeley Way West, Berkeley, CA USA; 4 School of Public Health, University of California, 2121 Berkeley Way, Room 5302, Berkeley, CA USA; 5 School of Public Health, UC Berkeley-UCSF Joint Medical Program, University of California, 2199 Addison St, 4th floor, Berkeley, CA USA

**Keywords:** Childhood, Obesity, Overweight, Vietnam, Young lives

## Abstract

**Background:**

Over recent decades, Vietnam has experienced rapid economic growth, a nutrition transition from the traditional diet to highly-processed and calorie-dense foods and beverages, and an increasing prevalence of childhood overweight/obesity (ow/ob). The goal of this study is to describe the patterns of ow/ob in a longitudinal sample of Vietnamese children from ages 1 to 8, and the sociodemographic and behavioral factors associated with ow/ob at age 8.

**Methods:**

This study is a secondary data analysis of a geographically-representative, longitudinal cohort of 1961 Vietnamese children from the Young Lives Cohort Study from 2002 to 2009. Thirty-one communities were selected with oversampling in rural communities, and children age 1 were recruited from each community using simple random sampling. Surveys of families and measurements of children were collected at child ages 1, 5, and 8. Our specified outcome measure was childhood ow/ob at age 8, defined by the World Health Organization’s thresholds for body-mass-index (BMI) for age Z-scores. Associations between early and concurrent socio-behavioral factors, childhood nutrition and physical activity variables were analyzed using STATA 15. Bivariate and multivariable analyses were completed utilizing logistic regression models.

**Results:**

The prevalence of ow/ob increased from 1.1% in both sexes at age 1 to 7% in females and 13% in males at age 8. Bivariate analyses show greater likelihood of ow/ob at age 8 was significantly associated with early life sociodemographic factors (at age 1), male sex (OR = 2.2, 1.6–3.1), higher wealth (OR = 1.1–1.4), and urban residence (OR = 4.3, 3–6). In adjusted analyses, ow/ob at age 8 was associated with early nutrition practices at age 5, including frequent consumption of powdered milk (OR = 2.8, 1.6–4.6), honey/sugar (OR = 2.7, 1.8–4.1), prepared restaurant/fast foods (OR = 4.6, 2.6–8.2), and packaged sweets (OR = 3.4, 2.3–4.9). In addition, breastfeeding for 6 months or longer was protective against obesity at age 8 (OR = 0.3, 0.1–0.9).

**Conclusions:**

We found that increased consumption of powdered milk, honey/sugar, packaged sweets, and prepared restaurants/fast foods are associated with childhood ow/ob. In contrast, breastfeeding for 6 months or longer was protective against childhood ow/ob. These findings suggest that public health programs and campaigns aimed to prevent childhood ow/ob in Vietnam should target early feeding practices.

**Supplementary Information:**

The online version contains supplementary material available at 10.1186/s12889-021-10292-z.

## Background

Over recent decades, many low- and middle-income countries (LMICs), such as Vietnam, have experienced rapid economic growth, particularly in urban areas [1]. This pattern has contributed to nutritional disparities and a “double-burden of child malnutrition,” with persistent undernutrition and increasing overweight/obesity (ow/ob) [2]. The prevalence of overweight and obesity among children less than 5 years of age has more than doubled, from 2.6% in 2000 to 5.9% in 2017 in Vietnam, estimated using World Health Organization’s Child Growth Standard between 2000 and 2017 [3].

Child obesity is associated with chronic health problems during childhood and increased risk for adult obesity and chronic diseases, including type 2 diabetes, hypercholesterolemia, cardiovascular disease, fatty liver, sleep apnea, and musculoskeletal disorders [4–6]. Furthermore, early childhood-onset obesity is associated with increased risk of premature mortality compared to adult-onset obesity [7–9]. In Vietnam, a case-control study from 2017 in Hanoi with children 6–11 years of age found that children with obesity tend to have more visceral fat than subcutaneous fat [10]. This difference is associated with increased levels of blood triglycerides and hypertriglyceridemia [10], which leads to increased risk of cardiovascular disease later in life.

Studies attribute the high prevalence of child obesity worldwide to economic globalization, driving a complex array of environmental and behavioral changes – primarily a “nutrition transition” from traditional diets high in carbohydrates and vegetables to diets with more animal products, ultra-processed foods, and high in fats and sugar [11–13]. This is also seen in Vietnam, along with other factors that have been linked to childhood obesity, including decreased physical activity due to greater availability of motor vehicle transportation, and increased sedentary screen-based entertainment [14, 15]. These economic and behavioral changes, and increasing child obesity, predominately impact urbanized areas and higher social economic strata [16–18].

Though there has been a surge in the number of cross-sectional studies exploring the prevalence and causes of ow/ob in Hanoi and Ho Chi Minh City, there is a lack of longitudinal studies that follow a cohort of children to examine the factors that contribute to this rapid increase in ow/ob. In order to develop effective strategies to prevent child obesity in Vietnam and its health consequences, there is a need for longitudinal data identifying the specific community-, family-, and child-level risk factors associated with childhood obesity across a broader span of early childhood years.

The goal of this study is to describe the patterns of ow/ob in a cohort sample of Vietnamese children at ages 1, 5, and 8. Additionally, our goal is to examine early (variables at ages 1 and 5) and concurrent (variables at age 8) socio-behavioral risk factors associated with child ow/ob at age 8.

## Methods

### Data source

This was a secondary data analysis of a longitudinal dataset of Vietnamese children and their families in the Young Lives Cohort Study (YLCS) (https://www.younglives.org.uk). YLCS is an international longitudinal research project spearheaded by the University of Oxford in collaboration with research institutes, universities and NGOs in four study countries. YLCS followed a large multi-national cohort of 12,000 children for 15 years in Peru, Ethiopia, India, and Vietnam with the goal of providing researchers information on individual children, their families, and communities to explore causes and consequences of childhood poverty, health & nutrition, and impacts on child development. For the purposes of our study, we chose to focus on YLCS’s Vietnam data.

### Study design, population, and setting

YLCS’s Vietnam study sites were selected in 2001 using a sentinel site surveillance system sampling approach discussed in YLCS’s technical note [19]. This methodology is multistage, purposive and random sampling that randomizes households from a study site chosen based on predetermined criteria from YLCS. First, five provinces were selected to be geographically representative of regions from North, Central, and South Vietnam – Hung Yen, Lao Cai, Da Nang, Phu Yen, and Ben Tre. These five provinces were chosen to include urban, rural, and mountainous regions that also reflect the heterogeneity of ethnicity and religion in Vietnam’s populations. In alignment with YLCS’s intention to study causes and consequences of children poverty, provincial government staff helped researchers select sentinel sites in each province with the intention of using “over-poor sampling strategy” [19]. This strategy entailed selecting four communes in each province with two communes from the poor group, as ranked by the Provincial Committee for Population of each province, one from the average group, and one from above-average group. However, if a selected commune had a population of less than 6000 persons, a similar commune of the same poverty level was selected to ensure that 100 children could be enrolled from that sentinel site. In total, thirty-one communes were selected to form 20 sentinel sites, with fifteen communes from the lowest-income group (48%), nine communes from the average-income group (29%), and seven communes from the above-average income group (23%). In each commune, a door-to-door screening survey for children born between January 2001 and May 2002 was performed from April to June 2002 to produce a list of eligible children. The non-response rate, noted as refusals by caregivers, was less than 2% (a total of 36 refusals). From each sentinel site, 100 children were randomly sampled using simple random sampling [19].

### Data collection

**Fig. 1:**
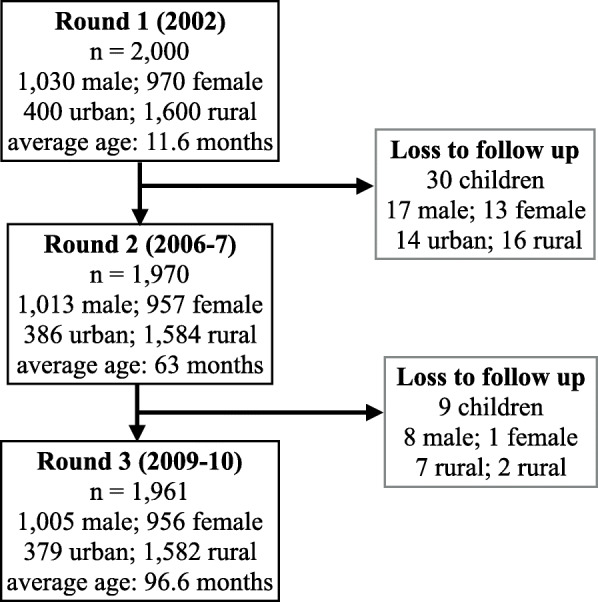
Cohort sample size and description by age

Three rounds of quantitative surveys of children and households were collected over 8 years: in 2002 (round 1, child age 1), 2006 (round 2, child age 5), and 2009 (round 3, child age 8). Of the 2000 children recruited in round 1, there was 98% follow-up, and the final sample upon completion of round 3 was 1961 children (Fig. 1).

### Anthropometric variables

Children were weighed and measured according to World Health Organization (WHO) standards. Body-mass-index (BMI) for age Z-score was calculated using WHO AnthroPlus [20]. The outcome variable was child ow/ob defined according to WHO thresholds [21]: For children under age 5 [22], overweight is BMI > 2 and < 3 Z-score, and obesity is BMI > 3 Z-score; For children ages 5–19 [23], overweight is BMI > 1 and < 2 Z-score, and obesity is BMI > 2 Z-score. Since the proportions of children with overweight and obesity were relatively small, we combined overweight and obese categories to produce the binary outcome variable of “overweight/obese” (ow/ob) for logistic regression.

### Variables

The outcome of interest in this study is ow/ob at age 8 as described in the anthropometric variables section above. Independent nutrition and physical activity variables were chosen based on causal pathways and associations previously supported by literature and available data from YLCS [24].

The only nutrition variable from age 1 is length of breastfeeding (less than 6 months, 6–12 months, and greater than 12 months). Beyond age 1, the questionnaire collected information at age 5 and 8 on frequency of consumption of powdered milk, milk/milk products, packaged sweets/snacks, honey/sugar, and food from restaurants/food stalls. For these variables in our bivariate analyses and parsimonious analyses (Table 3), the sample was divided into quartiles, based on reported frequency over the last 2 weeks. The two exceptions were for number of times a child ate per day at age 5 and 8, and frequency of consumption of powdered milk at age 8. For number of times a child ate per day, we chose 3 to be the reference since this would correspond with breakfast, lunch, and dinner. At age 8, because so few children drank powdered milk, it was only possible to analyze frequency in two groups—above and below the median—instead of quartiles.

For our extended multivariable analyses (Table 4), we found that the nutrition variables divided into quartiles as described above (including consumption of packaged sweets, restaurant/food stalls, sugar/honey, and powdered milk) did not work for these models because the sample cells of each quartile became too small for comparison. In order to observe the main overall effect and interaction of these nutrition variables, we split them into two groups down the median, instead of quartiles. Subsequently, we created new indicator variables that compared the children in the group above the median to the group of children who were below the median specifically for these analyses.

Physical activity variables collected at age 5 and 8 were not direct measurements of physical activity, so we chose mode of transportation to school, hours spent playing in the last week, and number of TVs owned in household as proxy variables. For mode of transportation to school, we used walking to school as a reference in comparison to being driven by motorbike. For the variables of hours spent playing in the last week and number of TVs owned per household, we divided the sample into tertiles.

The frequencies of nutrition and physical activity variables can be found in the supplemental appendix (Table 3.1).

### Statistical analysis

All statistical analyses were conducted in the statistical program STATA 15 (College Station, Texas). We divided the data by collection rounds. Within each round, the variables were grouped into child characteristics (including child sex, nutrition, and physical activity), maternal and household characteristics, and neighborhood characteristics.

The sociodemographic covariates were divided into child, household, and community characteristics. At the child level, we included sex, since male sex was positively associated with ow/ob. At the household level, we adjusted for wealth index [25] (a measurement of socioeconomic status calculated as an average of the housing quality index, consumer durables index and services index), since higher wealth index was positively associated with ow/ob. According to a technical report from YLCS, the wealth index was constructed based on previous work from the World Bank and Macro International’s wealth index cited in UNICEF’s Multiple Indicator Survey [26]. The average of the three indices discussed produces a score between 0 and 1, in which a higher score indicates a higher socio-economic status. At the community level, we adjusted for urban or rural site type, since urban location was positively associated with ow/ob.

To determine whether to model log odds as a linear function of a continuous variable or to create categories, we examined the LOWESS (Locally Weighted Scatterplot Smoothing) plots of the log odds for the nutrition and physical activity variables. If the resulting graph showed an approximately linear relationship, the variable was taken as continuous. If the graph did not show an approximately linear relationship, the variable was recorded into categories that were modeled with indicator variables, as supported by previous literature. Ow/ob at age 8 was a binary outcome.

We completed bivariate analyses using logistic regression to explore the associations of each independent nutrition and physical activity variable with the child’s ow/ob status at age 8. If the variable showed statistical significance (*p*-value < 0.05), we then included it in our adjusted model (adjusting for the covariates sex, wealth index, site type, and interaction between wealth index and site type) shown in the parsimonious model in Table 3.

We created two extended multivariable models to investigate the individual contribution of nutrition and physical activity variables in relation to each other and their associations with ow/ob at age 8, adjusting for the same sociodemographic covariates from the parsimonious models (sex, wealth index, site type, and interaction term between wealth index and site type). Model 1 examined earlier data collected at age 1 (round 1) and age 5 (round 2) found to be significantly associated with ow/ob at age 8 (round 3) in the adjusted model. Model 2 combined data collected at age 8 (round 3), age 5 (round 2), and age 1 (round 1) found to be significantly associated with ow/ob at age 8 (round 3) in the adjusted model.

## Results

### Descriptive results

The mean household size is 4.6 people, with 80% of the households located in a rural site (Table 1). Mothers had a mean age of 27, mean education of 7th grade, and average of 2 children. There was a roughly equal distribution of female and male children. One in 8 children was born prematurely. Nearly all children (98%) were breastfed, and most (93%) were breastfed for more than 12 months.

**Table 1:** Maternal and child characteristics at baseline (age 1)

Variables		Mean + SD or N (%) (*n* = 1961)
Household	**Household size**	4.6 + 1.4
	**Wealth index**	0.5 + 0.2
	**Site type**	
	*Urban*	400 (20)
	*Rural*	1600 (80)
Maternal	**Age of mother**	27.2 + 5.8
	**Highest maternal education (grade)**	6.8 + 3.8
	**Number of children born to mother**	1.9 + 1.2
Child	**Sex**	
	*Male*	1005 (51.2)
	*Female*	956 (48.8)
	**Child age (months)**	
	*Round 1*	11.6 + 3.2
	*Round 2*	63.0 + 3.8
	*Round 3*	96.6 + 3.8
	**Birthweight (grams)**	3100.2 + 446.7
	**Ever breastfed**	1893 (98.3)
	**Length of time breastfed (months)**	
	*0- < 6 months*	38 (1.9)
	*6–12 months*	96 (4.9)
	*12+ months*	1828 (93.2)
	**Born premature**	245 (12.5)
	**Prevalence of overweight & obesity**^a^	
	*Round 1*	22 (1.1)
	*Round 2*	165 (8.4)
	*Round 3*	199 (10.3)

^a^Overweight and obesity is defined using WHO’s definition. Less than 5 years of age, overweight is > 2+ SD BMI for age Z-score; obese > 3 + SD. Age 5–19, overweight is > 1+ SD; obese is > 2+ SD

**Fig. 2:**
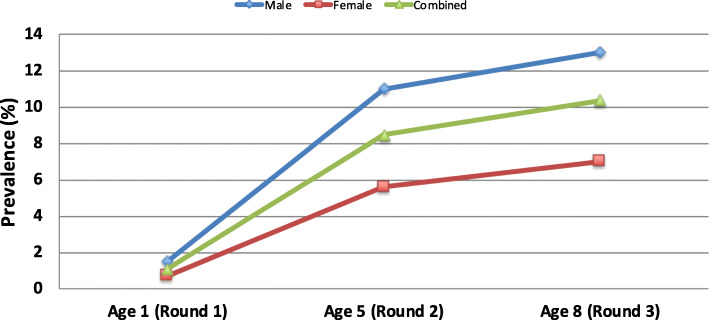
Prevalence of child overweight/obesity^1^ by age and sex ^1^Overweight and obesity is defined using WHO’s definition. Less than 5 years of age, overweight is > 2+ SD BMI for age Z-score; obese > 3 + SD. Age 5–19, overweight is > 1+ SD; obese is > 2+ SD.

The prevalence of childhood ow/ob increased with each round of data collection: age 1 (1.1%), age 5 (8.4%), and age 8 (10.3%). Using a Chi-square test and stratifying ow/ob by sex reveals that between age 1 and 5, there was statistical significance in rates of increase for ow/ob—by age 5, boys had twice the rate of ow/ob than girls, and this two-fold disparity was maintained from 5 to 8 years of age (Fig. 2).

Additionally, 50% of the children with ow/ob at age 1 continued to be ow/ob at age 5. And of the children with ow/ob at age 5, 65% continued to be ow/ob at age 8.

### Bivariate analysis: Sociodemographic and behavioral associations

Child ow/ob at age 8 was associated with numerous variables, including family sociodemographic, child characteristics, household characteristics, and child nutrition and physical activity characteristics (Appendix, Supplemental Table 2.1). Using bivariate analyses, the variables that were statistically significant were then added to the multivariable regression (section c) and multivariable models (section d).

The key associations in our bivariate analysis at age 1 was that breastfeeding for longer than 6 months was associated with decreased odds of ow/ob, compared to children who were breastfed for less than 6 months.

At age 5, ow/ob at age 8 was associated with owning a TV at home for household characteristics. For nutrition variables, the outcome was associated with being in the highest quartile for consumption of powdered/formula milk, packaged sweets, and prepared foods from restaurants/food stalls. Eating more than 6 times per day was also associated with higher odds of being ow/ob at age 8. As for physical activity variables that were statistically significant in the bivariate analysis at this age was being driven to school by motorbike, compared to walking. In contrast, spending 10 or more hours playing in the previous week was associated with decreased odds of ow/ob, compared with children who spent less time playing.

At age 8, nutrition factors associated with ow/ob at age 8 included being in the highest quartile for consumption of soft drinks, packaged sweets, and prepared foods from restaurants/food stalls in the last 2 weeks. Additionally, eating more than 6 times per day and having sugary drinks in the last 24 h were also associated with higher odds. Similar to age 5, transportation to school via motorbike was associated with ow/ob at age 8.

### Multivariable regression: Sociodemographic and behavioral associations

All odds ratios are reported with 95% confidence intervals. Using logistic regression, the sociodemographic variables significantly and positively associated (*p*-value< 0.001) with ow/ob at age 8 were male sex (OR = 2.2, 1.6–3.1), urban site (OR = 4.3, 3–6), and wealth index (Table 2).

**Table 2:** Sociodemographic associations with child overweight/obesity at age 8

Variables	Odds Ratio^a^ [95% CI]
Sex (Boy)	2.2 [1.6–3.1]***
Site type (Urban)	4.3 [3–6]***
Wealth index (5% increase)	
Urban WI	1.4 [1.3–1.6]***
Rural WI	1.1 [1.1–1.2]***

^a^The odds ratio for each variable is found after adjusting for the other

socio-demographic variables in this table

*p < 0.05; **p < 0.01; ***p < 0.001

Testing for interaction between our covariates, we found the association between ow/ob and wealth index was greater for urban (OR 1.4, 1.3–1.6) communities than rural (OR 1.1, 1.1–1.2). Therefore, we kept this interaction term in our models.

Additionally, we found an association between ow/ob and transportation to school via motorbike was greater for rural children (OR 1.13, 1.02–1.2) than urban children (OR 1.10, 1.002–1.22), with an overall p-value of 0.06. Since this difference is small and at the 100th decimal point, we did not keep this interaction term in our model.

Adjusting for sociodemographic variables (sex, site type, and wealth index), we examined associations between nutrition and physical activity variables at each round separately with ow/ob at age 8. Key findings that are potentially modifiable factors for future interventions are discussed in this section (Table 3).

**Table 3:** Parsimonious adjusted models (nutrition & physical activity variables in multivariable analyses, adjusted for sociodemographic variables)

	Variables	Age 1 OR [95% CI]	Age 5 OR [95% CI]	Age 8 OR [95% CI]
Nutrition Variables	**length of breastfeeding** *(ref is < 6 months)*			
	6–12 months	0.3 [0.1–0.9]*		
	> 12 months	0.4 [0.2–0.9]**		
	**# of times child ate/day** *(ref is 3 times)*			
	0–2 times		0.6 [0.3–1.1]	1.9 [0.2–18.8]
	4–5 times		0.8 [0.4–1.5]	1.5 [0.8–2.9]
	6 + times		2.4 [1.3–4.6]***	3.2 [1.6–6.8]***
	**restaurant/food stalls**^b^ *(ref is lowest quartile)*			
	2nd quartile		1.2 [0.6–2.5]	1.4 [0.8–2.4]
	3rd quartile		2.6 [1.4–4.7]***	1.8 [1.1–2.9]**
	4th quartile		4.6 [2.6–8.2]***	2.6 [1.5–4.3]***
	**powdered milk**^b,c^ *(ref is lowest quartile)*			
	2nd quartile		3.1 [1.8–5.3]***	
	3rd quartile		2.4 [1.4–4.1]***	
	4th quartile		2.8 [1.6–4.6]***	1.8 [1.1–3.0]*
	**milk/milk products**^b^ *(ref is lowest quartile)*			
	2nd quartile		1.4 [0.8–2.6]	0.9 [0.5–1.5]
	3rd quartile		2.4 [1.4–4.0]***	1.2 [0.7–2.0]
	4th quartile		3.9 [1.6–4.2]***	1.1 [0.6–1.8]
	**packaged sweets/** **snacks**^b^ *(ref is lowest quartile)*			
	2nd quartile		1.4 [0.9–2.3]	2.1 [1.4–3.2]***
	3rd quartile		2.3 [1.6–3.5]***	2.4 [1.6–3.8]***
	4th quartile		3.4 [2.3–4.9]***	3.5 [2.4–5.1]***
	**honey/sugar**^b^ *(ref is lowest quartile)*			
	2nd quartile		1.5 [1.0–2.3]	0.9 [0.6–1.4]
	3rd quartile		2.0 [1.3–3.2]***	1.0 [0.5–2.0]
	4th quartile		2.7 [1.8–4.1]***	1.5 [0.9–5.1]
Physical Activity Variables	**mode of transportation** *(ref is walking)*			
	riding bike		1.3 [0.8–2.1]	1.6 [0.9–2.7]
	motorbike		3.4 [2.1–5.0]***	3.8 [2.5–5.9]***
	**hours child spent playing in last week** *(ref is < 6 h)*			
	6–10 h		1.6 [1.0–2.7]	1.0 [0.6–1.3]
	10h hours		1.2 [0.7–2.1]	0.6 [0.5–1.1]
	**number of TVs owned in household**			
	*(ref is 0)*			
	1 TV		2.0 [0.9–4.2]	2.4 [0.7–8.0]
	2+ TV		2.8 [1.1–7.0]	4.9 [1.4–17.4]*

^a^All variables are adjusted for sex, site type, wealth index, and interaction term of wealth and site type

^b^The sample was divided into four equally sized groups or quartiles based on consumption within the last 2 weeks

^c^The sample for powdered milk at age 8 was divided into two equally sized groups above and below the median since it was not possible to divide it into quartiles

*p < 0.05; **p < 0.01; ***p < 0.001

#### Nutrition variables (associated with ow/Ob at age 8)

##### Age 1

At age 1, children who breastfed for 6 months or longer had approximately 1/3 the odds (OR = 0.3, 0.1–0.9) of becoming ow/ob at age 8, compared to children breastfed for less than 6 months.

##### Age 5

At age 5, being in the highest quartile for consumption of honey/sugar, milk/milk products, powdered milk or packaged sweets/snacks was significantly associated with higher odds of ow/ob. Children in the highest quartile for consumption of honey/sugar or powdered milk had nearly 3 times the odds of ow/ob (OR = 2.7, 1.8–4.1 and OR = 2.8, 1.6–4.6 respectively), and those in the highest quartile for consumption of milk/milk products had almost 4 times the odds of ow/ob (OR = 3.9, 1.6–4.2), compared to children in the lowest quartile of consumption. Children in the highest quartile for frequency of eating out at restaurants/food stalls had nearly 5 times the odds of ow/ob (OR = 4.6, 2.6–8.2) while those in the highest quartile for packaged sweets/snacks had 3 times the odds of ow/ob (OR = 3.4, 2.3–4.9), compared to children in the lowest quartile. Lastly, children who ate 6 times or more in the last 24 h had over twice the odds of ow/ob (OR = 2.4, 1.3–4.6), compared to children who ate fewer times.

##### Age 8

At age 8, children who consumed powdered milk more frequently than the median had nearly twice the risk of ow/ob (OR = 1.8, 1.1–3.0), compared to children who drank powdered milk less frequently. Additionally, children who ate 6 times or more daily had over 3 times the odds of ow/ob (OR = 3.2, 1.6–6.8), compared to children who ate fewer times. Children in the highest quartile for frequency of eating in restaurant/food stalls or packaged sweets/snacks had nearly 3 times the odds of ow/ob (OR = 2.6, 1.5–4.3 and OR = 3.5, 2.4–5.1, respectively), compared to children in the lowest quartile.

#### Physical activity variables (associated with ow/Ob at age 8)

Children driven to school via motor vehicle had over 3 times the odds of ow/ob at age 5 (OR = 3.4, 2.1–5.0) and age 8 (OR = 3.8, 2.5–5.9). There was a negative association between ow/ob and the number of hours a child spent playing in the last week, at ages 5 and 8. However, the variable lost statistical significance after adjusting for sociodemographic factors. In lieu of a variable for the number of hours children spent watching television, the number of televisions in a household was used as a proxy. Children with 2 or more televisions in their homes at age 8 had nearly 5 times the odds of ow/ob (OR = 4.9, 1.4–17.4), compared to children with no television.

### Multivariable models

**Fig. 3:**
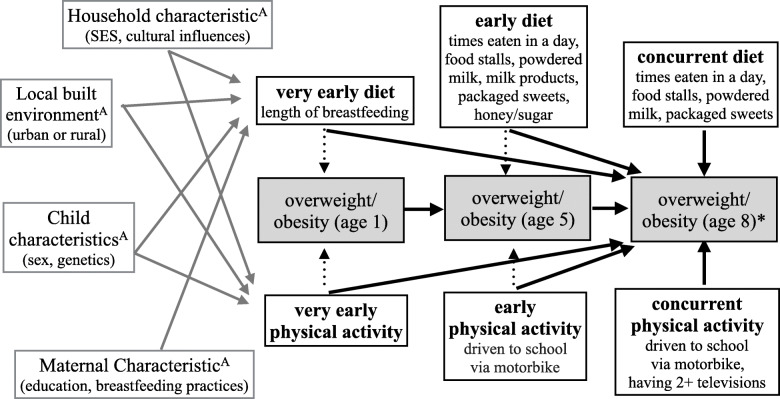
Summary of findings from parsimonious adjusted models^1^ ^1^This figure summarizes our findings from our adjusted parsimonious models adjusting for sex, site type, wealth index, and interaction term between wealth index and site type. ^A^The domains in the gray boxes continue to affect childhood overweight and obesity in the same direction in subsequent rounds (ages 5 and 8). ^*^Ow/ob at age 8 is the measured outcome. Dotted arrows were not direct measured outcomes.

Table 4 shows the two models (as described in statistical analysis section above) used to control for multiple factors, in addition to sociodemographic variables, to identify contributions of each factor from our adjusted parsimonious model. Figure 3 summarizes the findings of the sociodemographic, nutrition, and physical activity variables associated with childhood obesity in this sample.

**Table 4:** Extended multivariable models of risk factors associated with child overweight/obesity at age 8

Variables	Model 1 (Age 1 & 5) Early factors OR [95% CI]; *n* = 726	Model 2 (Age 1, 5, & 8) Combined Factors OR [95% CI]; *n* = 1004
**Social Demographics**		
wealth index	1.1 [1.0–1.2]	1.0 [0.9–1.1]
sex (boy)	3.2 [1.9–5.3]***	2.4 [1.4–4.3]***
site type (urban)	1.4 [0.2–9.9]	0.2 [0.0–3.1]
interaction term (wealth index x site type)	1.0 [0.9–1.2]	1.0 [0.8–1.2]
**Round 3**		
number of TVs (1+)^a^		2.6 [0.8–2.3]
transportation (motorbike)^b^		3.5 [1.8–6.7]***
times eaten (6+ times)^c^		1.8 [0.9–3.5]
packaged sweets^d^		1.1 [0.9–1.3]
restaurant/stalls^d^		1.5 [0.7–3.1]
milk^d^		2.5 [0.9–4.1]
**Round 2**		
transportation (motorbike)^b^	1.8 [1.1–3.3]*	1.4 [0.6–3.1]
times eaten (6+ times)^c^	1.6 [0.8–3.3]	2.3 [0.9–6]
powdered milk^d^	1.5 [0.8–2.7]	1.4 [0.7–2.8]
packaged sweets^d^	2.2 [1.3–3.5]**	1.9 [1.1–3.3]*
sugar/honey^d^	1.2 [0.6–2.0]	1.3 [0.8–2.4]
**Round 1**		
length of breastfeeding^e^	0.5 [0.1–2.2]	0.4 [0.1–1.5]
mother’s education^f^	1.7 [0.8–3.5]	2.1 [1.2–3.9]*

^a^ Number of TV household owned 1+ is compared to not owning any TV

^b^Mode of transportation via motorbike to school is compared to children who walk to school

^c^Number of times eaten (6+) is compared to baseline of eating 3 times a day

^d^Nutrition variables in these models are divided into two equally sized groups – above and below the median. The children who are above the median of consumption are compared those below the median of consumption

^e^Length of breastfeeding is greater than 6 months compared to those who breastfed for 6 months or less

^f^Mother’s education is comparing mothers with grade 10 or higher to those with less years of education

*p < 0.05; **p < 0.01; ***p < 0.001

#### Sociodemographic variables

The only sociodemographic variable consistently associated with ow/ob across the two models was male sex—males had 2 to 3 times the odds of ow/ob compared to females. In model 2, children whose mothers had high school or higher education had greater odds of ow/ob (OR = 2.1, 1.2–3.9).

#### Nutrition variables

In both models 1 and 2, children in the highest quartile for frequency of consumption of packaged sweets had higher odds of ow/ob in model 1 (OR = 2.2, 1.3–3.5) and model 2 (OR = 1.9, 1.1–3.3), compared to those in the lowest quartile.

#### Physical activity variables

Children driven to school via motor vehicle had higher odds of ow/ob in model 1 (OR = 1.8, 1.1–3.3) and model 2 (OR = 3.5, 1.8–6.7).

## Discussion

In the longitudinal YLCS, following nearly 2000 children at 1, 5 and 8 years of age from 2002 to 2009, the prevalence of childhood ow/ob increased with age. The increase in obesity rate was steepest from age 1 to 5 (1.1 to 8.4%), with a slower rate of increase from age 5 to 8 (8.4 to 10.3%). At age 1, the prevalence of ow/ob stratified by sex was not statistically significant, but by age 5 and 8, rates were almost double for boys when compared to girls. Children in urban sites had 4 times the odds of being ow/ob, compared to rural sites. Furthermore, ow/ob was significantly associated with wealth index in both urban and rural sites, but the odds ratio was greater for urban sites than rural sites. Higher maternal education was also associated with greater odds for child ow/ob. We also found that half of the children with ow/ob at age 1 continued to be ow/ob at age 5. And of the children with ow/ob at age 5, more than half continued to be ow/ob at age 8.

The findings on prevalence of ow/ob at all ages in this sample from 2009 are lower when compared to the more recent cross-sectional studies of similar age groups in Vietnam. A 2016 survey of 2677 children age 3–6 years old in Hanoi shows the prevalence of ow/ob to be 16.7 and 4.5%, respectively [27]. In the same year, a survey of children ages 7–9 years in Ho Chi Minh City using International Obesity Task Force cutoffs found the prevalence of overweight to be 30% and obesity to be 12.3% [18]. Ho Chi Minh City and Hanoi are both metropolitan areas, which may explain the higher rates of ow/ob in these studies. Meanwhile, the YLCS sampling methodology favored data from more rural communes. However, urban versus rural sampling may only partly explain this difference, as the more recent data may be consistent with the current trend of increasing rates of childhood ow/ob in Vietnam [28].

While other studies have identified numerous contributors to child obesity across community-, family- and child-level factors [29–31], we focused on child nutrition and physical activity factors that are potentially modifiable. In our bivariate and multivariable logistic regression models, the early childhood and concurrent nutritional risk factors associated with ow/ob at age 8 were frequent consumption of packaged sweets/snacks, powdered milk, milk/milk products, honey/sugar, restaurant/food stall food, and eating 6 or more times per day; while breastfeeding for more than 6 months was a protective nutrition factor. The key physical activity risk factors for ow/ob were motor vehicle transportation to school, and having two or more televisions in the home.

This study’s findings of the prevalence of ow/ob, and the associated sociodemographic and behavioral factors, are consistent with findings of other cross-sectional studies in Vietnam [17, 18, 32, 33] and other LMICs [34–36]. In LMICs, higher rates of child ow/ob for families with greater wealth, particularly in urban areas, are likely attributable to globalization, economic growth and marketing-driven changes in child feeding practices (consumption of a greater quantity and frequency of calorie-dense food and beverages) [37, 38] and reduced physical activity (due to urban traffic with less opportunity to safely walk to school and play outdoors, and greater use of sedentary screen-based entertainment) [29, 39–41].

As LMICs’ economic growth continues, the burden of ow/ob will likely shift from families in the higher economic strata to lower economic strata [42]. In a recently published paper, Templin et al. extrapolates from existing data to predict that the percent change in overweight prevalence among the relatively poor in Vietnam from 2016 to 2040 will be 80–100% [43]. This projection is alarming, as the Vietnamese government is still struggling to address stunting and wasting for children under 5 years of age from families of lower wealth [44].

Aside from wealth difference in association with ow/ob at age 8, we also found sex difference, in which more boys were ow/ob at age 5 and age 8. In Vietnam, boys may have higher ow/ob odds due to historical and cultural male-gender preferences [45], possibly leading families to devote more resources to boys in regards to feeding practices. Furthermore, this difference may be a consequence of culturally embedded preferences for “thinner” girls and “bigger” boys [46].

Longer breastfeeding was found to be a protective nutrition factor, consistent with previous literature from many other countries [47–49], possibly since longer breastfeeding may preclude excessive bottle-feeding and inappropriate complementary feeding practices that predispose the child to obesity in early childhood [50]. Although high rates of breastfeeding are reported in this study, bottle-feeding and supplemental feeding practices are not recorded. Other studies show high rates of mixed feeding [51] and bottle-feeding [52] in Vietnam – especially supplemental feeding with sweetened liquids and foods [53] that is associated with increased risk of child obesity [54]. Children of mothers with higher education may have greater risk of ow/ob because educated mothers may be working outside the home, curtailing the duration of breastfeeding, and leaving the child’s caregiver with money to buy “treats” such as snacks or sugary drinks [55, 56]. Furthermore, the common practice of giving children sweets as a reward leads children to associate sugary foods and beverages with positive emotions, and reduces preference for non-sugary options such as vegetables, unsweetened milk, and water [57–59].

In Vietnam, economic growth is a major driver of increased availability of ultra-processed snack foods, fast foods, and sugar-sweetened milk. The 2016 USDA Foreign Agriculture Service Report reported that Vietnam is the 13th largest export market for food and beverages from the United States, and that Vietnam’s sizable young population, rising middle-class incomes, and female labor force participation may be driving consumerism and preferences for processed, packaged products and fast food chains [60]. With the increase in accessibility of Western-style fast food [61] and dietary pattern changes due to increased consumption of processed foods [62], Vietnamese children now have access to more processed snacks and fast foods. This may explain our findings that children who eat snacks, eat more frequently, and eat out in food stalls/restaurants have higher odds of being ow/ob. Aside from snacking, eating more frequently is dependent on caretakers’ feeding practices. In a cross-sectional study on parents’ feeding practices and their association to childhood obesity*,* Do et al. found children of mothers in rural area of Hanoi were more likely to eat larger amounts of food, with high intake of fatty foods and protein, when compared to children of mothers in the urban environment of Hanoi [63]. The paper hypothesized that this may be a result of “pressure to eat” feeding practices of mothers in rural area stemming from fear of malnutrition, and consequently leading to both increased amounts of food and higher intake of fatty foods [63].

In this study, we also found that children who drink powdered milk and milk products have higher odds of being ow/ob at age 8. In a recent qualitative study by Do et al., mothers acknowledged that one way to improve children’s diets is to switch from milk with sugar to milk without sugar [55]. While milk and powdered milk have some nutritional benefits, the sugar and other additives may be a significant contributor to excess weight gain. There is substantial evidence that consumption of sugar-sweetened beverages, including sugary milk, contributes to the epidemic of child ow/ob [64, 65]. However, sugary milk consumption has been understudied, and unfortunately the YLCS did not specify in the questionnaire if milk contained sugar or not.

Aside from nutrition, physical inactivity and sedentary behaviors have been identified as major risk factors contributing to increase in ow/ob in LMICs [41]. Since the dataset did not include direct measures of daily physical activity, children who were being driven to school via motorbike was used as a proxy for decreased physical activity, with findings suggesting that children who were driven to school have higher odds of being ow/ob in YLCS. From interviews with parents, Trang et al. found that lack of pathways and dangerous traffic were among the reasons many parents did not let their children walk or cycle to school in Ho Chi Minh City [17]. Decreases in walkability of cities in Vietnam [66], in combination with increases in screen time [67], are leading to an increase in sedentary behaviors amongst children in Vietnam, which subsequently contribute to the rising trend of ow/ob.

Overall, this study supports and adds to previous findings that early childhood parenting practices around child nutrition and physical activity—and the environmental and cultural context—play a crucial role in contributing to child ow/ob, and may be important targets for interventions to prevent child ow/ob. Additionally, these interventions should aim to reach groups that are at greatest risk of developing child ow/ob, specifically children who are boys, from families of higher economic strata, and living in urban settings.

### Study strengths and limitations

The strengths of this study include utilizing the YLCS, a large geographically-representative 8-year longitudinal cohort dataset, with very low attrition (1.5%), enabling investigation of a wide variety of sociodemographic and behavioral risk factors for child overweight/obesity at ages 1, 5, and 8 – which, before this study, had not been done. Our study focused on potentially-modifiable nutrition variables—identified as very early factors (age 1), early factors (age 5) and concurrent factors (age 8)—associated with child ow/ob at age 8. We utilized parsimonious models adjusting for sociodemographic factors and extended models adjusting for very early life, early and concurrent sociodemographic and behavioral factors.

The limitations of this study include the secondary analysis of an existing dataset that is over 10 years old and not specifically designed to include all variables relevant to child obesity, especially daily physical activity. Furthermore, the dataset does not include structural variables such as food supply and access, which is a function of structural constraints in childhood obesity [68]. BMI-for-age is a simple and useful, but imperfect, proxy for identifying child adiposity and related health risks [69]. The main limitation of the extended models is that multi-collinearity may have reduced the statistical significance for significant variables in the prior adjusted models. The significant associations identified in this study do not prove causation of the outcomes, although the temporality of pre-existing factors before the outcomes in this longitudinal study adds further support for the hypothesis, beyond the findings of cross-sectional studies. In regards to physical activity variables assessed in our analyses, these were not direct measurements of physical activity, but were instead proxy variables used in substitution as YLCS’s surveys did not address children’s physical activity levels. Finally, while the pro-rural sampling in the YLCS facilitated urban vs. rural analysis, weights were not provided to correct for this; therefore, it is not possible to accurately generalize our findings to the population nationwide in Vietnam. In comparison to Vietnam’s 2002 Demographic and Health Survey and 2002 Vietnam Housing Living Standard Survey, households in YLCS on average were slightly poorer and owned fewer assets [19].

## Conclusions

This longitudinal, geographically-representative study in Vietnam found a high prevalence of early childhood ow/ob, increasing dramatically between 1 and 8 years of age. Sociodemographic risk factors included male sex, higher family wealth, higher maternal education, and urban location. Nutritional risk factors included frequent meals and frequent consumption of milk/milk products, sweets, packaged snacks, and food from restaurants/food stalls, while longer duration of breastfeeding was protective. Physical activity risk factors included being driven to school and having multiple televisions in the home.

There is an urgent need for policies and interventions to prevent child obesity in Vietnam—particularly focused on early childhood from age 1–5 years—with support for healthy parenting practices regarding nutrition and physical activity. These include limiting the marketing of unhealthy products (including sugar-sweetened milk) to children and parents, prohibiting the sale of unhealthy foods and beverages in and around schools and health facilities, increasing public media and education of parents in maternal-child health programs and preschools regarding longer breastfeeding, limiting obesogenic foods/drinks and screen time, and increasing physical activity.

## Supplementary Information

**Additional file 1 Table S2.1**. Bivariate associations with overweight and obesity at Round 3 (age 8). **Table S3.1**: Frequencies of Nutrition & Physical Activity Variables.

## Data Availability

The datasets generated and/or analyzed in the current study are available in the Young Lives Cohort Study repository through UK Data Service, https://www.younglives.org.uk. Datasets from UK Data Service are open to public access through three tiers of access: tier 1 – open (de-identified data with low risk of disclosure), tier 2 – safeguarded (de-identified data but at risk of disclosure due to linkage to other data), and tier 3 – controlled (data that may be identifiable and are at high risk of disclosure). For this study, we registered and were approved by UK Data Service for tier 1 access, which is open to the general public.

## Abbreviations

Ow/ob: Overweight and obese
LMIC: Low- and middle-income countries
BMI: Body mass index
WHO: World Health Organization
YLCS: Young Lives Cohort Study
