# Supplementary material for: Early childhood factors associated with obesity at age 8 in Vietnamese children: The Young Lives Cohort Study
Source: BMC Public Health. 2021 Feb 5;21:301. doi: 10.1186/s12889-021-10292-z (PMC7866641; doi:10.1186/s12889-021-10292-z)
Supplement: Supplementary file 1 — Additional file 1 Table S2.1. Bivariate associations with overweight and obesity at Round 3 (age 8). Table S3.1: Frequencies of Nutrition & Physical Activity Variables. [file 12889_2021_10292_MOESM1_ESM.docx]

# Supplemental Appendices

| **Table 2.1 - Bivariate associations with overweight and obesity at Round 3 (age 8)** | | | | |
| --- | --- | --- | --- | --- |
|  | **Variable** | **OR [95% CI]** | **P-value compared to reference** | **Overall P-value^1^** |
| **ROUND 3 (Age 8)** | | | |  |
| **Nutrition** | **Estimated quantity of soft drinks bought & consumed in last 2 weeks** |  |  |  |
|  | *0 times (reference)* |  |  | 0.004 |
|  | *<5 times* | 1.2 [0.8- 2.1] | 0.304 |  |
|  | *>5 times* | 2.1 [1.4-3.4] | 0.001 |  |
|  | **Estimated quantity of packaged sweets bought & consumed in last 2 weeks** |  |  |  |
|  | *0 (reference)* |  |  | <0.001 |
|  | *1-2 times* | 2.1 [1.4-3.2] | <0.001 |  |
|  | *3-5 times* | 1.7 [1.3-2.9] | 0.003 |  |
|  | *>5 times* | 1.6 [1.1- 2.7] | 0.029 |  |
|  | **Estimated quantity of powdered milk/formula** |  |  |  |
|  | *0 (reference)* |  |  | <0.001 |
|  | *1 more times* | 3.2 [1.8- 4.3] | <0.001 |  |
|  | **Estimated quantity of milk/milk products bought & consumed in last 2 weeks** |  |  |  |
|  | *0 times (reference)* |  |  | <0.001 |
|  | *1-5 times* | 2.2 [1.2-3.7] | 0.006 |  |
|  | *5-15 times* | 2.7 [1.8-4.2] | <0.001 |  |
|  | *>15 times* | 3.8 [2.5-5.9] | <0.001 |  |
|  | **Estimated quantity of prepared foods bought & consumed in last 2 weeks** |  |  |  |
|  | *0 times (reference)* |  |  | <0.001 |
|  | *1-5 times* | 0.9 [0.5-1.9] | 0.996 |  |
|  | *5-10 times* | 3.6 [2.2-6.2] | <0.001 |  |
|  | *10-15 times* | 4.2 [2.6-6.6] | <0.001 |  |
|  | *>15 times* | 7.5 [4.7-11.9] | <0.001 |  |
|  | **Did child eat sweet, honey, or sugary drinks in the last 24 hours?** | 1.8 [1.2-2.6] | 0.001 | 0.001 |
|  | **Number of times child ate in the last 24 hours** |  |  |  |
|  | *3 times (reference)* |  |  | <0.001 |
|  | *4-5 times* | 2.5 [1.3-4.6] | 0.003 |  |
|  | *6-7 times* | 7.8 [4.1-14.8] | <0.001 |  |
| **Physical Activities** | **Hours child spent playing/general leisure time in the last week (during the week)** |  |  |  |
|  | *<6 hours (reference)* |  |  | 0.008 |
|  | *6-10 hours* | 1.0 [0.7-1.4] | 0.889 |  |
|  | *>10 hours* | 0.5 [0.4-0.8] | 0.004 |  |
|  | **Transportation to School** |  |  |  |
|  | *Walking (reference)* |  |  | <0.001 |
|  | *Bike* | 1.3 [0.8-2.2] | 0.261 |  |
|  | *Motorbike* | 7.3 [5.0-10.9] | <0.001 |  |
|  | **Number of Television in household** |  |  |  |
|  | *0 (reference)* |  |  | <0.001 |
|  | *1 TV* | 3.5 [1.2-7.2] | <0.001 |  |
|  | *2+ TV* | 8.7 [6.5-12.3] | <0.001 |  |
| **ROUND 2 (Age 5)** | | | |  |
| **Household Characteristics** | **Owns a Television** | 5.4 [2.7-10.7] | <0.001 | <0.001 |
|  | **Number of Television in household** |  |  |  |
|  | *0 (reference)* |  |  | <0.001 |
|  | *1 TV* | 4.3 [2.2-0.6] | <0.001 |  |
|  | *2+ TV* | 22.7 [10.7- 48.2] | <0.001 |  |
| **Nutrition** | **Number of times child ate within the last 24 hours** |  |  |  |
|  | *3 times (reference)* |  |  | <0.001 |
|  | *<3 times* | 2.4 [0.3-20.4] | 0.407 |  |
|  | *4-5 times* | 1.9 [1.1-3.3] | 0.029 |  |
|  | *6+ times* | 6.7 [3.8-11.8] | <0.001 |  |
|  | **Estimated quantity of powdered/formula milk bought & consumed in last 2 weeks** |  |  |  |
|  | *Lowest quartile* |  |  | <0.001 |
|  | *2^nd^ quartile* | 3.5 [2.1-5.8] | <0.001 |  |
|  | *3^rd^ quartile* | 5.2 [3.3-8.2] | <0.001 |  |
|  | *Highest quartile* | 9.2 [6.0-14.2] | <0.001 |  |
|  | **Estimated quantity of packaged sweets bought and consumed in the last 2 weeks** |  |  |  |
|  | *Lowest quartile* |  |  | <0.001 |
|  | *2^nd^ quartile* | 1.3 [0.8-2.1] | <0.001 |  |
|  | *3^rd^ quartile* | 2.4 [1.6-3.6] | <0.001 |  |
|  | *Highest quartile* | 3.4 [2.4-4.9] | <0.001 |  |
|  | **Estimated quantity of sugar/honey bought and consumed in the last 2 weeks** |  |  |  |
|  | *Lowest quartile* |  |  | <0.001 |
|  | *2^nd^ quartile* | 2.3 [1.5-3.5] | <0.001 |  |
|  | *3^rd^ quartile* | 2.5 [1.7-3.8] | <0.001 |  |
|  | *Highest quartile* | 1.8 [1.1-2.8] | 0.011 |  |
|  | **Estimated quantity of milk/milk products bought and consumed in the last 2 weeks** |  |  |  |
|  | *Lowest quartile (reference)* |  |  | <0.001 |
|  | *2^nd^ quartile* | 1.4 [0.7-2.6] | 0.265 |  |
|  | *3^rd^ quartile* | 2.4 [1.4-4.0] | 0.001 |  |
|  | *Highest quartile* | 3.9 [1.3-4.3] | <0.001 |  |
|  | **Estimated quantity of prepared foods (restaurant/food stalls) bought and consumed in the last 2 weeks** |  |  |  |
|  |  |  |  |  |
|  |  |  |  |  |
|  |  |  |  |  |
|  | *Lowest quartile (reference)* |  |  | <0.001 |
|  | *2^nd^ quartile* | 1.2 [0.6-2.4] | 0.615 |  |
|  | *3^rd^ quartile* | 2.6 [1.4-4.6] | 0.002 |  |
|  | *Highest quartile* | 4.6 [2.6-8.2] | <0.001 |  |
| **Physical Activity** | **Transportation to School** |  |  |  |
|  | *Walking (reference)* |  |  | <0.001 |
|  | *Bike* | 1.2 [0.7-1.8] | 0.509 |  |
|  | *Motorbike* | 6.3 [4.3-9.3] | <0.001 |  |
|  | **Hours child spent playing/general leisure time in the last week (during the week)** |  |  |  |
|  | *<6 hours (reference)* |  |  | <0.001 |
|  | *6-10 hours* | 0.5 [0.3-0.7] | 0.002 |  |
|  | *>10 hours* | 0.3 [0.2-0.6] | <0.001 |  |
| **ROUND 1 (Age 1)** | | | |  |
| **Household Characteristics** | **Wealth index** | 1.3 [1.2-1.3] | <0.001 | <0.001 |
| **Mother’s Characteristics** | **Mother’s age (years)** |  |  |  |
|  | *>=24 to <=28 (reference)* |  |  | <0.001 |
|  | *<24* | 0.3 [0.2-0.5] | <0.001 |  |
|  | *>28* | 1.1 [0.7-1.4] | 0.754 |  |
|  | **Mother’s education** |  |  |  |
|  | *No education (reference)* |  |  | <0.001 |
|  | *Primary school (grade 1-5)* | 4.6 [1.4-15.4] | 0.011 |  |
|  | *Middle school (grade 6-9)* | 5.6 [1.7-17.9] | 0.004 |  |
|  | *High school (10-12)* | 17.0 [5.2- 55.9] | <0.001 |  |
|  | *University or Vocation school* | 43.4 [13.2- 42.9] | <0.001 |  |
| **Nutrition** | **Length of breast feeding (m)** |  |  |  |
|  | *0-<6 months (reference)* |  |  | <0.001 |
|  | *6-12 months* | 0.2 [0.0-0.4] | <0.001 |  |
|  | *>12 months* | 0.2 [0.1-0.3] | <0.001 |  |
| *^1^Overall P-value = Pearson’s chi-square test* | |  |  |  |

**Table 3.1: Frequencies of Nutrition & Physical Activity Variables**

|  | Variables | Age 1 | Age 5 | Age 8 |
| --- | --- | --- | --- | --- |
| Nutrition Variables | **Length of breastfeeding**  *(ref is <6 months)*                  6-12 months                  >12 months | 1.94%  4.89%  93.17% |  |  |
|  | **# of times child ate/day**  *(ref is 3 times)*                 0-2 times  4-5 times  6 + times |  | 15.93%  19.8%  37.20%  26.45% | 25.72%  0.80%  57.97%  16.30% |
|  | **restaurant/food stalls**^B^  *(ref is lowest quartile)*                  2nd quartile                  3rd quartile                  4th quartile |  | 32.54%  19.23%  25.89%  22.34% | 54.42%  14.92%  16.10%  14.56% |
|  | **powdered milk**^B,C^  *(ref is lowest quartile)*                  2nd quartile                  3rd quartile                  4th quartile |  | 81.88%  6.04%  6.24%  5.84% | 56.80%  43.20% |
|  | **milk/milk products**^B^  *(ref is lowest quartile)*                 2nd quartile                  3rd quartile                  4th quartile |  | 30.89%  19.96%  26.08%  23.07% | 27.84%  24.18%  23.88%  24.10% |
|  | **packaged sweets/**  **snacks**^B^  *(ref is lowest quartile)*                 2nd quartile                  3rd quartile                  4th quartile |  | 54.72%  14.26%  15.99%  15.03% | 59.68%  14.31%  11.96%  14.05% |
|  | **Honey/sugar**^B^  *(ref is lowest quartile)*                 2nd quartile                  3rd quartile                  4th quartile |  | 41.32%  21.57%  19.70%  17.41% | 33.72%  34.34%  7.31%  24.63% |
| Physical Activity  Variables | **Mode of transportation**  *(ref is walking)*                  Riding bike  motorbike |  | 40.44%  32.46%  27.10% | 49.13%  28.10%  22.77% |
|  | **Hours child spent playing in last week**  *(ref is <6 hours)*                  6-10 hours  10+ hours |  | 49.78%  24.53%  25.64% | 44.07%  29.96%  25.96% |
|  | **Number of TVs owned in household**  *(ref is 0)*                  1 TV  2+ TV |  | 18.99%  73.9%  7.11% | 9.96%  73.49%  9.95% |

*^A^All variables are adjusted for sex, site type, wealth index, and interaction term of wealth and site type.*

*^B^The sample was divided into four equally sized groups or quartiles based on consumption within the last 2 weeks.*

*^C^The sample for powdered milk at age 8 was divided into two equally sized groups above and below the median since there were not enough children drinking powdered milk at age 8.*
